# Law-Enforcement Challenges, Responses and Collaborations Concerning Environmental Crimes and Harms in Bangladesh

**DOI:** 10.1177/0306624X20969938

**Published:** 2020-11-05

**Authors:** Sarker Faroque, Nigel South

**Affiliations:** 1 Police Staff College, Bangladesh; 2 University of Essex, UK

**Keywords:** Bangladesh, environmental crimes, law enforcement, police, environmental courts

## Abstract

As Kailemia observes, “environmental crimes are an area of increasing concern, not only because of [their] globalized nature” but because these crimes have “impacts beyond the capacity of criminal justice systems of most states to comprehend or address.” How then can criminal justice agencies, particularly in the global south, respond to national and transnational environmental challenges? This paper takes the case of Bangladesh and outlines examples of anthropogenic activities that are destroying biodiversity and polluting the environment in this country. It then situates these crimes and harms in the context of environmental law, and the work of police and environment agencies, in Bangladesh. The paper concludes with a consideration of future options for environmental law enforcement in Bangladesh.

## Introduction

Environmental crimes increasingly draw global attention as wide-spread understanding of their short-term impacts and long-term consequences grows. Violations of environmental laws and regulations might be local but the effects that follow may be felt long distances away. Hence, such crimes are acknowledged as “a serious problem worldwide in different forms, with some of them being among the most profitable criminal activities in the world. The most common crimes against the environment are connected with the unlawful exploitation of wild fauna and flora, pollution and waste disposal” (Sustainability for All, 2018). This is a problem affecting the whole planet—but it is particularly the global south that has been vulnerable to external and internal environmental exploitation and damage caused by, for example, extractive industries, or government sponsored infrastructure mega-projects (Brisman et al., 2018; Deb, 2020; Goyes, 2018; Goyes et al., 2017; White, 2018). Bangladesh is no exception to this. As Yardley (2013: Section-A:1) observes, “Environmental damage usually trails rapid industrialization in developing countries. But Bangladesh is already one of the world’s most environmentally fragile places, densely populated yet braided by river systems, with a labyrinth of low-lying wetlands leading to the Bay of Bengal. Even as pollution threatens agriculture and public health, Bangladesh is acutely vulnerable to climate change, as rising sea levels and changing weather patterns could displace millions of people and sharply reduce crop yields.”

The conventional, broad categories of environmental crimes found in various jurisdictions are summarized by White (2011, p. 3) and these are also found in Bangladesh: “unauthorized acts or omissions that are against the law and therefore subject to criminal prosecution and criminal sanctions; crimes that involve some kind of cross-border transference and an international or global dimension; and crimes related to pollution (of air, water, and land); crimes against wildlife (including illegal trade in ivory as well as of live animals); and illegal fishing . . . .” In Bangladesh, pollution, trafficking in wildlife (including critically endangered species), deforestation, dumping of electronic, industrial and domestic waste, and illegal fishing, are all common and well-known environmental crimes. Severe air, water, and noise pollution are threatening human health, ecosystems and economic growth in the country and, as shown below, are the subjects of media and political attention.

This paper takes the case of Bangladesh and provides examples of anthropogenic activities that are destroying biodiversity and polluting the environment. It then situates these crimes and harms in the context of, first, environmental law and regulation, and then, second, in relation to the work of the police and specific environment agencies, in Bangladesh. The paper follows the work of Pink and White (2016a, 2016b) and Jones and Honorato (2016), in aiming to provide a preliminary overview of capacity and collaboration, as well as obstacles to cooperation, in Bangladesh. However, as Kailemia (2018) observes, “environmental crimes are an area of increasing concern, not only because of [their] globalized nature” but because these crimes have “impacts beyond the capacity of criminal justice systems of most states to comprehend or address.” A central question here then is to ask how criminal justice and conservation agencies, particularly in economies of the global south, can respond to national and transnational environmental challenges?

## Environmental Crimes and Harms, and Enforcement and Collaborative Responses in Bangladesh

According to Pink and White (2016b, pp. 3–4) we can approach the breadth of these challenges by thinking of environmental crimes in terms of a “continuum” of ways of conceptualizing them, whether by devising and applying “strict legal definitions” of “crimes,” or employing “broader harm perspectives” that are capable of incorporating, for example, those “unauthorized” acts or omissions that violate the law and that might attract criminal justice system responses (see also: Situ & Emmons, 2000, p. 3; South, 2014). Certain acts can also be addressed in term of intentions and outcomes, for example, whether they are *meant* to cause “harm to ecological and/or biological systems” with the aim of achieving some advantage (Clifford & Edwards, 198, p. 126) or whether they are “crimes of omission” (Huisman & van Erp, 2013), ignorance or denial.

The complexity of environmental crimes and harms makes criminal justice and law enforcement responses difficult. If ever there were a field of illegal, legal but harmful, or simply anti-social, activity in need of a combined response and collaborative efforts, it is this. Pink and White (2016b, pp. 8–9) outline the bases for such collaboration in terms of a series of questions “commonly referred to by law enforcement and regulatory staff as either the ‘5w’s and 1 h” or the “six loyal servants”: these are “who, what, where, when, why, and how”? As will become clear when considering the Bangladesh case, it is useful to consider the prospects for collaboration in relation to the following scoping exercise:

- identify who the relevant partners/stakeholders are,
- determine what the focus (or main purpose) of the collaboration is,
- decide where the collaboration/s might be coordinated from or take place,
- agree when the collaboration will commence and might conclude,
- establish why collaboration is considered beneficial, and
- discuss how the collaboration will most likely proceed (Pink & White, 2016b, p. 9).

Beyond the narrow remits of domestic enforcement agencies, small scale partnerships and larger collaborations increasingly involve wider groups of stakeholders and interest groups while intergovernmental bodies and non-governmental organizations (NGOs) are also leaders or facilitators of collaborative interventions (Pink & White, 2016b, p. 6; see also: Interpol & CITES, 2007; Kangaspunta & Marshall, 2009; White, 2012).

## Biodiversity, Environmental Challenges and Anthropogenic Impacts in Bangladesh

Bangladesh is a small but densely populated country with rich biodiversity and natural ecosystems.^1^ It is one of the most ecologically significant and biologically diverse landscapes in the world, with natural ecosystems that include several types of forests, freshwater wetlands and distinctive coastal and marine features. Some protected areas, for example, the Sundarbans, have international World Heritage site status; other, less well-known but important habitats include the haors (wetland basins) in northeastern Bangladesh and tropical evergreen forests in the Chittagong Hill Tracts. As described below, all are currently threatened in different ways.

In fact, Bangladesh faces many direct threats to its biodiversity related to anthropogenic behaviors and attitudes towards the environment. Continuing growth, economic development and an increasing population mean many of these threats will intensify leading to (1) encroachment on protected areas; (2) degradation of forests and wetlands; (3) damage resulting from infrastructure development; (4) unsustainable and/or illegal exploitation of land resources; (5) unsustainable and/or illegal fishing practices; (6) negative changes in hydrological regimes; (7) increased pollution; and (8) impacts of invasive species (USAID, 2016). The prospects are summarized in one newspaper report (Ahmed, 2016, p. 7) reviewing the major collection of essays on *Contemporary Environmental Challenges in Bangladesh* (Rahman, 2015):

*Degradation of the natural environment and its impact on human lives is now visible all over the world. As a densely populated country with limited natural resources, the situation in Bangladesh is even more precarious. Environmental pollution, especially . . . linked to soil, water and air, [has] emerged as a big challenge to sustainable development of the country.*

Haque (2017) notes that the World Health Organization has ranked Bangladesh fourth among 91 countries with the worst urban air quality, and Islam (2018, p. 32) summarizes as follows: “environmental contamination in Bangladesh is not a theory, it’s a way of life. At present air pollution has reached a dangerous point. According to the Department of Environment on 22 November 2017 the air pollution index of Dhaka city stood at 269. This is similar to the air pollution index of Delhi. Steady economic growth in our country has created many environmental challenges particularly in urban and industrial areas.” As the World Bank (2018) and Bangladesh national newspapers have reported, the country has experienced very high levels of environmental pollution with annually high rates of associated morbidity and mortality. In 2015:

*Bangladesh saw around 234,000 deaths, including 80,000 in urban areas, due to environmental pollution and related health risks . . ., making it one of the worst affected countries in the world . . . .Some 18,000 lives and 578,000 years of potential life were lost in Dhaka city in* 2015 *– the second least livable city in the world, showing the urgency to immediately address the city’s environmental issues. 58 percent of air pollution are caused by illegal brick kilns, 10 percent by vehicles, 20 percent by construction activities, and the rest by various other factors, including industries. (The Daily Star, 17th September 2018a, p. 1).*

More specifically, wastes from food production industries, growing urbanization, problems stemming from lack of efficient management of solid, clinical and e-wastes, river pollution, the results of over-use of pesticides, adulteration of food, and the harmful impact of coal-fired kilns used for producing bricks to meet the demand in construction and infrastructure^2^—all these provide a glimpse into the present state of environmental challenges in the country (The Daily Star, 2018a).

A wide range of environmental crimes and harms can therefore be identified in Bangladesh, from those driven by structural changes to the economy, to more individualized, often traditional but updated, forms of poaching and wildlife crime, land use that causes degeneration of agricultural land, misuse and depletion of forest resources (e.g., by hill cutting), and damage to wetlands and fisheries. Poaching, for example, can be very profitable, especially when dealing in rare species such as tigers (Kamal, 2019; Saif and MacMillan, 2016; The Daily Star, 2015). However, in Bangladesh (as elsewhere) environmental crimes and harms may not be given much priority or are not seen as a matter of urgency or importance, they may be denied, or if acknowledged may not be considered “crimes” resulting from “intentional behavior” or “criminal negligence” (South, 2016). This is partly because the violation of environmental laws and regulations that might be committed today may only have impacts that are felt or understood in many years. In searching for remedies and responses, it is increasingly argued that we need to re-evaluate day-to-day practices as citizens, as workers, as parents and families, as consumers, and as members of communities, to be more aware of the significance of day-to-day impacts on the environment and their cumulative effects (Agnew, 2013; Brisman & South, 2014).

### Economic Power, Humanitarian Crisis and Ecological Consequences

In Bangladesh—as elsewhere—ordinary people commit environmental crimes without serious thought about their behavior and its effects, often as an expression of self-interest or competitive consumerism (Agnew, 2013; Brisman & South, 2014), while the treadmill of production and pursuit of profit underpin and spur corporate harms to the environment (Ruggiero & South, 2013a, 2013b; Stretesky et al., 2014). For example, such behavior is causing considerable damage and destruction in the Sundarbans (a mosaic of islands that is home to many unique terrestrial, aquatic, and marine habitats, ranging from micro- to macro-flora and fauna) (Bisschop et al., 2020, p. 618). The Sundarbans is not only a site of importance for Bangladesh but also for global conservation as home to various endangered species, including the Royal Bengal Tiger, Ganges and Irrawaddy dolphins, estuarine crocodiles, and also the critically endangered endemic river terrapin, and the largest mangrove forest in the world. The area is a UNESCO World Heritage site yet as the secretary of the National Committee to Protect Oil, Gas, Mineral Resources, Power and Ports, currently leading opposition to the Rampal power plant being built close to the Sundarbans, has stated:

*The Sundarbans, a magnificent and unique ecosystem of the world, faces an existential question today with a coal power plant to be set up at Rampal. . . .The Sundarbans has a special role in the fight against climate change effects. The government is signing deals over climate change, bringing in funds but destroying the country’s strongest shield in this battle – the Sundarbans. We can’t afford to lose the Sundarbans.* (Muhammad, 2016, p. 1)

The opposition to these damaging proposals has gained international momentum leading to the use of social media and, in November 2018, the mobilization of global protest networks to highlight the issue. The climate change protest group 350.org supported this campaign by reporting that the protests were designed to “save the world’s largest mangrove forest from a proposed coal power plant” (350.Org, 2018).

Other areas of crucial bio-diversity and ecological importance in Bangladesh are also under threat due to different pressures. For example, a report from UNDP, UN Women and the Ministry of Environment, Forests and Climate Change (2018) has reported on the significant environmental impacts of the major inward migration of refugees fleeing genocidal discrimination in Myanmar (BBC News, 2020), noting that “Since the influx in August 2017, coupled with the host community and refugees from past influxes, the crisis affected population is now almost 1.5 million in Cox’s Bazar, creating massive pressure on the already dilapidated environment. . .” (The Daily Star, 2018b). The need to provide resources for a *humanitarian* crisis is now creating an *environmental* crisis in coastal areas such as “Teknaf, Saint Martin Island and Sonadia Island and two more restricted areas near these camps—Himchhari national park and Teknaf sanctuary. The proposed Inani National Park is also at risk” (UNDP and UN Women, 2018, p. 31). An immense variety of species—around 1,156 species of plants and animals—inhabit the latter area, including many already listed as endangered such as elephants, deer, Indian wild cats, and wild hogs.

The refugee emergency has accelerated the anthropogenic impacts of land reclamation and human encroachment, leading to threats to biodiversity and pollution of the environment in sensitive and previously protected areas. As Shuvo (2018, p. 13) elaborates, “about 4,000 acres of hilly tracts have already been cut down to construct camps for Rohingyas in Bangladesh. Including the surrounding area of those hills, the occupied area is about 10,000 acres in total.” Fuel for cooking and heating water must be sourced from the surrounding woodlands—the impact is considerable if “around 50,000 kg of firewood is needed” every day.

Even in the face of such data, trends and major news stories however, unlike other criminal activities, environmental crimes and harms do not receive a high priority response. This is not to say Bangladesh has neglected to put in place strong environmental and natural resources management policies and regulations. In fact, the country has a range of laws directed at protection of the environment, a national environment ministry, and new special courts that deal with environmental cases. These laws, policies and initiatives are discussed below. Yet despite all this, pollution is rising, not falling, largely because of the political and economic power of industry and corporate interests, as well as governmental commitment to growth. Bangladesh suffers particularly acutely because of this combination of factors.

## Law and Environmental Crime in Bangladesh

The Police Act of 1861 (Section 34), describes some actions related to environmental pollution which are punishable and police officers are empowered to arrest people who are involved in these offenses. Regarding the protection and improvement of the environment and biodiversity, Article 18A of the 1972 Constitution of the People’s Republic of Bangladesh, defines the responsibility of the state as being to “endeavor to protect and improve the environment and to preserve and safeguard the natural resources, bio-diversity, wetlands, forests and wildlife for present and future citizens.” A wide range of laws and other provisions are available to employ in cases of environmental crime and crimes against animals. These include Statutory Laws: The Cruelty to Animals Act, 1920; the Animal Disease Act, 2005; the Animal Slaughter and Meat Control Act, 2011; and the Bangladesh Wildlife Conservation and Security Act, 2012. As the brief examples below illustrate, the legal provisions of the latter Act are frequently employed by the police, under the terms of Chapter 8 authorizing investigation and seizure in cases of illegally hunted, acquired and captured wild animals, their illegal purchase, sale and import/export, the killing of certain animals, and the contravention of regulatory provisions.

The Constitution of Bangladesh (Articles 31 and 32) enshrines the “right to life and personal liberty” as a fundamental right and the Supreme Court has resolved that the “right to life” includes the “right to a healthy environment” (see also Al Mahdi, 2015; Mohammad, 2012). The Bangladesh Police are therefore empowered by the constitution, state and Supreme Court, to ensure that these rights to a clean and healthy environment are upheld. However, obstacles to the exercise of police powers and to collaboration between agencies may follow from political and economic opposition, as well as from unhelpful lines of accountability and direction. For example, criminal justice agencies are responsible to the Ministry of Home Affairs but the Department of Environment and the Bangladesh Forest Department are both directed by the Ministry of Environment, Forest and Climate Change. This is particularly important to an understanding of why the apparently progressive development of introducing Environmental Courts has yielded poor impacts and excluded significant sources of complaint from its remit. This situation is discussed in more detail below.

Bangladesh is signatory to various international conventions, treaties and protocols (ICTPs) regarding the conservation and protection of the environment (Islam, 1996) and the current National Biodiversity Strategy and Action Plan has been developed in order to “fulfill the commitment of Bangladesh towards implementing the three objectives of the Convention on Biological Diversity (CBD): conservation of biodiversity, the sustainable use of its components, and fair and equitable sharing of the benefits arising out of the utilization of genetic resources” (DoE, n.d., p. xiii). Nonetheless, according to the Environmental Performance Index Rankings (Yale Center et al., 2018), Bangladesh came 179th among 180 countries listed.

Shuvo (2018, p. 13) provides a valuable summary of current regulations and legal capacity for responses to environmental challenges broadly and with specific reference to the Rohingya refugee crisis. “Article 18(A) of the Constitution of the People’s Republic of Bangladesh talks about the protection and improvement of environment and biodiversity” with around “210 laws relating to the environment and over 30 policies, strategies and action plans pertinent to environmental administration.” The Environment Conservation Act (section 5), 1995 may be applied in response as it contains a provision to create what is called an Ecologically Critical Area (ECA). This provision has been used in the past, for example, in 1999 when the government declared that eight areas, including Cox’s Bazar and the Teknaf Peninsula, should be designated as ECAs. Shuvo also notes the relevance of the 1927 Forest Act in relation to forest conservation and management, particularly important in a country such as Bangladesh which has low forest availability and density. The ongoing processes of deforestation, increasing population growth and accompanying demands, have inevitable impacts on the size of forest land—and this erosion is now being accelerated to meet the needs of the refugees. Laws relating to wildlife conservation such as the Bangladesh Wildlife (Preservation) Order, 1973, are also applicable here as the clearing of forest cover is disrupting habitats and lifecycles, and even leading to deaths of, various vulnerable wild species.

**Figure 1.**
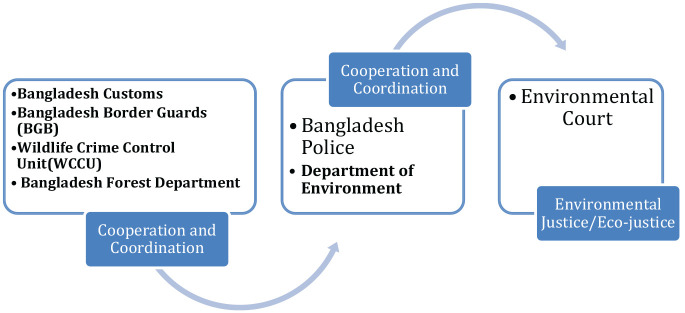
Agencies responding to environmental crime and harm in Bangladesh.

Despite all this, as Faruque (2018, p. 7) argues Bangladesh has myriad laws and regulations conforming to international guidelines to protect its environment, however it faces a significant challenge in managing compliance due to a lack of necessary resources and technical capacity within law enforcement agencies. The central components of the system of responses are illustrated in Figure 1.

The Bangladesh police are the key agency, while Forestry officials and officers have played an increasingly important role. One particularly significant development in the system of responses to environmental crime in Bangladesh has been the introduction of specially designated Environmental Courts to settle environmental disputes and apply environmental laws. This is an important innovation given the infancy of such initiatives globally (Pring & Pring, 2009). The operation and contribution of these courts is discussed below following a review of environmental policing in Bangladesh.

## Policing in Bangladesh and the Role of the Police in Environmental Crime Management

The Bangladesh Police force is the main law enforcement agency in the country and dates back to the Police Act of 1861. The organization has been modernizing and reforming to embrace problem-solving and community-oriented approaches after past criticism of performance and accountability (Commonwealth Human Rights Initiative, 2008; International Crisis Group, 2009). As elsewhere, the national police service in Bangladesh has responsibility for responding to criminal activities (investigation, apprehension of offenders, providing evidence for the criminal courts). However, a more contemporary problem-solving approach has emphasized the need for specific kinds of responses for specific kinds of problems. One-size-fits-all policing is not appropriate for problem-solving and intelligence-led policing and this is particularly the case in relation to environmental crime policing (Brisman & South, 2015; Nurse, 2015). Examples of three intelligence-led operations concerning hunting or collection of endangered species for the purposes of trafficking (Nurse & Wyatt, 2020; Sollund, 2019) are given below.

*Case 1*: On 20th September 2017, in the Uttara zone of the Dhaka Metropolitan Police area, police arrested five smugglers in possession of a substantial number of tortoises which, along with a baby crocodile, were recovered alive. The list of seized animals included: 67 small, medium and large sized live Hamilton type tortoises (weight 1–1.5 kg); 70 live Star type tortoises (length approximately between 8 and 10 cm); 455 live Balu river type tortoises (length approximately between 8 and 12 cm); 64 turtles (length approximately in between 09 and 18 cm); and 1 live baby crocodile (length approximately 08 and 12 cm). The case was dealt with under Section 25(B)(D) of the Special Powers Act 1974 (Smuggling or attempting to smuggle prohibited goods without paying tax or customs duties).

The resulting investigation found that:

◽ the apprehended perpetrators were engaged in smuggling or trafficking tortoises, crocodiles and other wild animals or wild species out of Bangladesh to overseas markets such as Singapore, Bangkok and Malaysia and had been doing so and thereby evading tax and customs duties, for several years.
◽ they planned to smuggle the seized animals using a route by air.
◽ communications with foreign nationals were uncovered and provided evidence regarding smuggling of tortoises, crocodiles and other wild animals. This intelligence was processed and circulated to Interpol through the Bangladesh National Crime Bureau (NCB), the agency of the Bangladesh Police that liaises and collaborates with INTERPOL and similar external police agencies.
*Case 2*: Operation Lion and Leopard, carried out in Jessore District on 3th November 2017, led to the arrest of five perpetrators involved in smuggling wild animals using a Prado Land Cruiser. The rescued animals included: two baby lions in one wooden box; two baby leopards in one wooden box. The land cruiser was seized by police. Prosecution followed under the Wild Animal Preservation and Protection Act 2012 which can result in fines or jail terms as punishment.

The resulting investigation found that:

◽ the apprehended perpetrators were engaged in transnational smuggling of wild animals or wild species.
◽ but significantly, Bangladesh was not the original source of the smuggled animals. This was a case of transit smuggling in which the perpetrators had acquired the animals from others operating across the porous border with India and then planned to smuggle these onward to other nations.
*Case 3*: In Operation Zebra, also in Jessore District, on 9th May 2018, police arrested four perpetrators and recovered nine zebras (eight found alive and one dead). This case was also pursued under the Wild Animal Preservation and Protection Act 2012.

All cases support the findings of a report from the US State Department that has “listed Bangladesh among the countries which are a major source, transit point or consumer of smuggled wildlife products and their derivatives” (The Daily Star, 2017).

## Inter-Agency Collaboration

In Bangladesh, the recent challenges of environmental crimes and harms have encouraged inter-agency cooperation with positive signs of benefits following from this. Bangladesh Police, Customs, the Forest Department especially the Wildlife Crime Control Unit (WCCU) are increasingly finding ways to work together. Collaborations have also involved external partners such as the INTERPOL Crime Unit which, for example, organized a conference on “Tortoise and Turtle Trafficking” at the Police Staff College for members of the Bangladesh Police, Customs Intelligence, Forest Department and Rapid Action Battalion (RAB). This multi-agency meeting provided a platform for future cooperation and actions in responding to environmental crimes in Bangladesh, especially wildlife crime. The meeting was also a means of developing connections with representatives from India, Malaysia and Thailand, to form a transnational platform aimed at wildlife trafficking in South and South-East Asia and urge greater integration of efforts regarding criminal investigation and apprehension. Bangladesh police have also benefited from international partnerships with bodies such as Interpol and the International Consortium on Combating Wildlife Crime. Working with the latter, Bangladesh was the site of the first in-country trial of the ICCWCs Wildlife and Forest Crime Analytic Toolkit (ICCWC, 2012), which “resulted in 40 targeted recommendations to improve the effectiveness of Bangladesh’s law enforcement and preventative responses to wildlife and forest crime (UNODC, 2013)” (Scanlon & Farroway, 2016, p. 87).

Pink and White (2016b, pp. 5–6) suggest that “Responding to environmental crime primarily falls to enforcement and regulatory agencies within government, whether at the national, subnational, or local level. In most parts of the world, the main response agencies are police agencies, customs and border protection agencies, and environmental regulatory agencies. These can be considered the ‘three core agencies’ of environmental law enforcement.” Organizationally this can present opportunities but also create barriers. In Bangladesh, the police fall under the authority of the Ministry of Home Affairs and this provides a clear remit and line of accountability in relation to most matters of law, order and security. The subjects of environmental policing and environmental justice are broad however and, as Pink and White indicate, are engaged with by a range of responsive bodies. The importance of two further agencies introduces a parallel and to some extent competing system. The Department of Environment, and the Bangladesh Forest Department, both fall under the authority of the Ministry of Environment, Forest and Climate Change. As indicated above, the Forest Department takes a role in relation to leadership on fighting wildlife crime and has a Wildlife Crime Control Unit (WCCU). This Unit collaborates with the police, particularly the National Crime Bureau, on operational activities against wildlife trafficking. Separately however, it is the Department of Environment that articulates with the Environmental Courts and it is plain clothes inspectors from this department who are responsible for investigation of offenses relating to environmental pollution and similar environmental harms. As described above, the police are more frequently involved in the investigation of wildlife trafficking cases under the Wild Animal Preservation and Protection Act 2012 and the Special Powers Act, 1974.

## Environmental Courts in Bangladesh

According to White (2013, p. 268) the growth of specialized courts focused on environmental matters reflects “expanding recognition of the need for procedural and substantive justice vis-à-vis environmental matters,” and Walters and Westerhuis (2013) among others, have noted the creation of such courts now extends across over 300 countries (Pring & Pring, 2009, p. 1). This list of countries includes Bangladesh which established its first environmental court in 2002 under provisions of the Environmental Court Act, 2000, succeeded by the Environmental Court Act 2010. Although this Act renewed the legal basis for the introduction of such specialized courts, as Sajal and Akhtaruzzaman (2016, pp. 215–217) argue, the Act did not recognize “the common people’s right of access to Environment Courts directly,” instead access—and hence control of the business of the courts—is a matter for officials of the Department of the Environment: “no Environment Court shall receive any claim for compensation under environmental law except on the written report of an Inspector of the Department of Environment (DoE).” Furthermore, the 2010 Act was intended to lead to the establishment of environmental courts in every district in order to curb the rate of increase of environmental crimes and enhance the efficiency of the trial process (Miah, 2015) but this too has not been implemented as well as it might have been. Progress in achieving this ambition has been slow and at present courts are operating in only a few locations, such as Dhaka (the capital city) and Chittagong (the major port city and second largest city). Even the specialized courts that have been established have been subject to criticism however regarding their failings due to confusions over jurisdiction, the lack of specialist knowledge and training of judges, and poor cooperation and standards of investigation among law enforcement agencies serving the courts. As Miah (2015) summarizes, the Act has not provided an efficient system for several reasons. First, the Act simply adds the responsibilities and functions of the environmental court to the general duties of existing district judges. As Miah asks, given the sizeable “backlog of cases in civil and criminal courts, how can a joint district judge perform the gigantic functions of civil, criminal and environmental courts?” Second, although the establishment of the courts recognizes that environmental offenses are of a “special nature involving scientific and technical implications of environmental violations” and “expert knowledge is specially required to determine the level or presence of pollution,” nonetheless, “the Environment Court Act, 2010 requires no such experts in the constitution of environmental courts.” Finally, given the courts are dependent upon written reports produced by Inspectors from the Department of the Environment, the system should be able to have reasonable expectations of the timeliness of delivery of such reports but “the ECA nowhere provides for any time-limit within which [an] investigation is to be concluded. As a result, the Inspectors frequently delay in submitting reports to the court” (Miah, 2015, p. 7).

Faruque (2018, p. 7) argues that the establishment of the Environmental Courts is a “proper step in the right direction for promoting access to environmental justice and providing remedies for violation of environmental laws in Bangladesh” but that “access to environmental justice is severely limited by the inherent weaknesses of the relevant laws and complicated procedures.” The courts are compromised by being unable to “operate independently of governmental influence and control” and because judges are often simply appointed from the ordinary court with no specialist knowledge or expertize about environmental matters. As Faruque suggests, it would seem reasonable that judges should receive “special training on environmental law and evolving environmental jurisprudence”; investigations and reports should be timely in order to expedite proceedings; and, given the contentious nature of many cases, “Provision should be made for the protection of witnesses in environmental cases” and “There should be special rules of evidence for dealing with environmental disputes.” As noted above, in terms of the desirability of maximizing capacity and encouraging collaboration, one major barrier to these goals is that the Courts are more closely linked to the Department of Environment than to the police.

## Discussion

It is possible to identify a number of gaps and weaknesses in current Acts, laws and policies that provide the framework for environmental crime policing, enforcement and justice in Bangladesh. First, penal provisions in current law are weak with a maximum 3-year jail sentence or relatively low financial penalty for poaching and trafficking in all wild animals except the killing of tigers and elephants. Second, there is insufficient public understanding of the frequency and diversity of wildlife crimes and a tendency to view these as a “normal,” “everyday” and relatively unimportant crime. Third, this extends to lack of awareness among the general public of the *ecological* and *economic* significance of wildlife crimes—for example, the monetary loss to some and criminal profits to others, and the implications of destruction of diversity and damage to the eco-balance of many areas. Fourth, this is a low-profile subject for law-enforcement and investigation—the number of cases investigated and prosecuted is relatively small, and although there is often a degree of media interest in these, journalists and the public are not very well informed about what is involved. Finally, the transnational nature of environmental/wildlife crimes presents many challenges to criminal investigation and law enforcement.

A number of important questions follow from this overview of environmental policing and justice in Bangladesh. For example, are relevant acts and policies in place? Are the existing acts and policies sufficient and strong enough to deal with these particular kinds of crimes? Do these acts and policies give the Bangladesh Police—and other bodies—the appropriate mandate to respond to all relevant crimes? What are the strengths and weakness that appear when working with other agencies to address environmental crimes?

As elsewhere, the value of collaboration and collaborative approaches has been noted in Bangladesh. International evidence (see e.g., essays in Pink & White, 2016a), at both domestic and international levels, shows that working with different stakeholders to face the challenges of environmental crime can be productive and Bangladesh has experienced some benefits from this, for example through engagement with Interpol. Collaboration across the core agencies in the Bangladesh response to environmental crime and harm, and between these and other key government response agencies, has seen encouraging signs but as Pink and White (2016b, p. 8) note, the success of collaboration can depend on many factors and need to be worked on. Among other elements this requires attention to the following: “valuing local knowledge and different perspectives, collaborative goal setting, sensitively challenging the taken-for-granted, trust, openness, and honesty (mutual respect), selecting the right people for the task, establishing the networks and relationships, and sharing of ideas, knowledge, and intelligence.”

## Conclusion

This paper has provided a brief overview of environmental crimes and harms, and the work of relevant responding police and other agencies, in Bangladesh. As described, Bangladesh has adopted a range of laws, policies and national strategies that address threats to and protection of biodiversity, several of which have been amended over the past decade to enhance enforcement efforts and powers. However, environmental laws and policies are not always well defined in general nor always well enforced on the ground. The reasons for this may include: “(1) poor institutional capacity; (2) lack of coordination among different agencies; (3) policy and information gaps; (4) lack of enforcement; (5) an inadequate and poorly managed system of protected areas; (6) corruption; (7) lack of political commitment; (8) lack of awareness; (9) climate and biophysical changes, and (10) lack of alternate livelihoods in sensitive habitats” (USAID, 2016).

Within the political and justice systems of any nation, environmental crimes may often be seen as of low importance and are not always accepted as “crimes” even though legal definitions apply. In Bangladesh, official documents such as the *Guide Book on Wildlife Law Enforcement in Bangladesh* (Dey & Rabbi, 2015) are endorsed by senior figures like the Chief Conservator of Forests of the Bangladesh Forest Department which is the agency responsible to the Department of Environment but the source of this endorsement highlights one of the organizational barriers to closer collaboration and cooperation between agencies within the country. There are issues and impediments that need to be acted upon in relation to collaboration and shared systems of accountability and responsibility. Achieving such aspirations is desirable but not necessarily all that easy to successfully accomplish. Invariably, say Pink and White (2016b, p. 9) different interests will be in play and “various partners and stake-holders” will reflect various viewpoints which prioritize or are influenced by matters of the economy, of conservation, or of environmental law enforcement (Wyatt, 2013, p. 163). As Bartel and Bricknell (2016, p. 228) observe, “building relationships between enforcement and regulatory agencies^3^. . . and the regulated community” can be difficult where each “may initially distrust the other but the reduction of social distance and creation of moral agreement carries great and potentially significant benefits.” Despite the continuing challenges, the prospects for responses and collaborations concerning environmental crimes and harms in Bangladesh are improving.

## Notes

1. For an overview and map, see, for example: BBC country profile https://www.bbc.co.uk/news/world-south-asia-12650940

2. Shaci and Price (2018) quote estimates that suggest this industry “employs more than one million people who churn out 23 billion bricks each year at some 7,000 kilns,” all burning coal.

3. The authors would add “researchers” here as an additional important constituency.
